# Body Mass Index and the Risk of Atrial Fibrillation: A Mendelian Randomization Study

**DOI:** 10.3390/nu14091878

**Published:** 2022-04-29

**Authors:** Mi Ma, Hong Zhi, Shengyi Yang, Evan Yi-Wen Yu, Lina Wang

**Affiliations:** 1 Key Laboratory of Environmental Medicine Engineering of Ministry of Education, Department of Epidemiology & Biostatistics, School of Public Health, Southeast University, Nanjing 210009, China; 13330591822@163.com (M.M.); 220203857@seu.edu.cn (S.Y.); evan.y.w.yu@gmail.com (E.Y.-W.Y.); 2 Department of Cardiology, Zhong Da Hospital, Southeast University, Nanjing 210009, China; 101005674@seu.edu.cn; 3 CAPHRI Care and Public Health Research Institute, School of Nutrition and Translational Research in Metabolism, Maastricht University, 6229 Maastricht, The Netherlands

**Keywords:** Mendelian randomization, BMI, atrial fibrillation, causal inference

## Abstract

Although observational studies have shown positive associations between body mass index (BMI) and the risk of atrial fibrillation (AF), the causal relationship is still uncertain owing to the susceptibility to confounding and reverse causation. This study aimed to examine the potential causality of BMI on AF by conducting a two-sample Mendelian randomization (TSMR) study. Methods: The independent genetic variants associated with BMI (*n* = 303) at the genome-wide significant level were derived as instrumental variables (IV) from the Genetic Investigation of Anthropometric Traits (GIANT) consortium consisting of 681,275 individuals of European ancestry. We then derived the outcome data from a GWAS meta-analysis comprised of 60,620 cases and 970,216 controls of European ancestry. The TSMR analyses were performed in five methods, namely inverse variance weighted (IVW) method, MR-Egger regression, the weighted median estimator (WME), the generalized summary data-based Mendelian randomization (GSMR), and the robust adjusted profile score (RAPS), to investigate whether BMI was causally associated with the risk of AF. Results: We found a genetically determined 1–standard deviation (SD) increment of BMI causally increased a 42.5% risk of AF (OR = 1.425; 95% CI, 1.346 to 1.509) based on the IVW method, which was consistent with the results of MR-Egger regression, WME, GSMR, as well as RAPS. The Mendelian randomization assumptions did not seem to be violated. Conclusion: This study provides evidence that higher BMI causally increased the risk of AF, suggesting control of BMI and obesity for prevention of AF.

## 1. Introduction

Atrial fibrillation (AF), the most common type of persistent clinical tachyarrhythmia, affected around 46.3 million individuals worldwide in 2016, according to The Global Burden of Disease project [1]. The current estimated prevalence of AF in adults is between 2% and 4% across the world [2], showing a 3-fold rise over the last 50 years based on data from the Framingham Heart Study [3]. It is biologically plausible that AF increases the risk of stroke and heart failure [4], resulting in the detrimental consequences of quality of life, disability, and even death. Moreover, given its high prevalence, AF is reported to be among the most expensive lifetime treatment of all diseases, which causes a considerable burden to the healthcare system [5].

As a surrogate marker of obesity, body mass index (BMI) has been a significant risk factor for obesity-related diseases, including AF. Several observational studies have reported a positive association between BMI and the risk of AF [6,7,8,9]. Nevertheless, it should be noted that the observed associations could not be well determined due to the limitations of conventional statistical methods, namely potential confounders either or both reverse causalities [10].

Mendelian randomization (MR) is a method that can be of support to resolve these limitations [11,12,13]. In such cases, MR eliminates systematic biases by selecting genetic variants associated with exposure as instrumental variables (IVs), analogous to randomized controlled trials (RCT), alleles are allocated randomly at conception according to Mendel’s second law [12]. Through that, confounders are randomly distributed throughout the population. Followed by identifying the common variants in outcome data, the fundamental guideline utilized in MR can be used to contemplate the effect of a suspected environmental exposure (e.g., BMI) on disease risk. Given credit to the primarily increased genome-wide association studies (GWASs) in the past decade, other studies already used MR to establish causation between common cardiovascular diseases and blood pressure [14], tumor necrosis factor levels [15], and other risk factors [16,17]; however, the evidence for the effect of BMI on AF is still limited.

To overcome the limitations of conventional observational studies and shed light on whether BMI is a cause of AF, we used a two-sample Mendelian randomization (TSMR) approach and estimated associations between single nucleotide polymorphisms associated with BMI and risk of AF based on two independent publicly available GWASs [18,19].

## 2. Materials and Methods

### 2.1. Data Source

The exposure variable data for the genetic variants associated with BMI was derived from a GWAS meta-analysis in the Genetic Investigation of Anthropometric Traits (https://portals.broadinstitute.org/collaboration/giant/index.php/GIANT_consortium_data_files., accessed on 28 April 2021) consortium (*n* = 681,275 individuals of European ancestry) [20]; similarly, the outcome variable data for AF was derived from a GWAS meta-analysis in six studies (The Nord-Trøndelag Health Study (HUNT), deCODE, the Michigan Genomics Initiative (MGI), DiscovEHR, UK Biobank, and the AFGen Consortium; http://csg.sph.umich.edu/willer/public/afib2018, accessed on 28 April 2021), which featured 60,620 AF cases and 970,216 controls of European ancestry [21].

### 2.2. Instrumental Variable Selection

The extracted genetic variants were selected as IVs to estimate causal effects of BMI on the risk of AF in accordance with the assumptions as follows: (1) being predictive of BMI, (2) being independent of confounders, and (3) no alteration of the outcome via an independent pathway other than BMI [22]. We firstly applied 751 SNPs associated with BMI at a genome-wide significance (*p* < 5 × 10^−8^). After pruning for linkage disequilibrium (LD) (r^2^ < 0.001; distance < 1000 kb), we obtained 507 independent SNPs. Each of the 507 BMI-associated SNPs was then examined the potential violations of the assumptions 2 and 3 based on the Phenoscanner database [23] (http://www.phenoscanner.medschl.cam.ac.uk/, accessed on 30 April 2021). Those SNPs, which significantly associated with any known risk factor (e.g., age, cardiovascular diseases, inflammation, dyslipidemia, and diabetes) for AF at a Bonferroni-correction *p* level (*p* < 9.9 × 10^−5^; 0.05/507) were excluded from the further analysis. In addition, the SNPs with other pleiotropic pathways (e.g., physical inactivity, smoking, and drinking) were also removed [24,25,26,27]. The remaining SNPs were aggregated with the AF GWAS database, removing 6 SNPs that were not included in the AF database, and 10 palindromic SNPs with intermediate allele frequencies. In addition, we used the RacialMR R package [28] to perform Cochran’s Q test on the remaining SNPs and excluded 43 heterogeneous SNPs. We also examined possible pleiotropy of the selected SNPs by using the MR pleiotropy residual sum and outlier (MR-PRESSO) test [29] and the HEIDI (heterogeneity in dependent instrument) approach based on the GSMR analysis [30], in which 7 SNPs were excluded. In total, we applied 303 valid SNPs as IVs (Supplementary Figure S1).

### 2.3. Statistical Analysis

#### 2.3.1. Mendelian Randomization Analyses

We employed the inverse variance weighted (IVW) method as the primary analysis to evaluate the causal effect between BMI and AF in our TSMR study. IVW calculates the exposure-outcome effect corresponding to each SNP using the Wald Ratio method, then performs a weighted linear regression with a forced intercept of zero. It achieved higher estimate accuracy and test power when IVs satisfied the three underlying assumptions [19]. To avoid the interference of unknown and unmeasurable confounders, we performed the MR-Egger regression (MR-Egger) [31], the weighted median estimator (WME) [32], the generalized summary data-based Mendelian randomization (GSMR) [30], and the robust adjusted profile score (RAPS) [33] to test the robustness of our results. 

A reliable MR-Egger assessment of the causal effect is possible if the strength of the genetic instrument does not correlate with the impact of the instrument on the outcome, known as the InSIDE (Instrument Strength Independent of Direct Effect) assumption. Additionally, it should be noted that the MR-Egger estimate of causal effect will be biased towards a null result due to the NOME (No Measurement Error) assumption violation [34]. Therefore, we calculated the $I_{GX}^{2}$ statistics for assessing NOME violation and used simulation extrapolation (SIMEX) [35] to adjust this potential bias when the $I_{GX}^{2}$ value is lower than 0.9. 

WME provided a consistent estimate of causality, even when up to 50% of genetic variants were invalid. GSMR analysis extends the SMR method using all the top associated SNPs at a genome-wide significance level for the exposure as IVs to test causality. Furthermore, unlike other methods, it accounts for both possible LD between SNPs and the sampling errors in the estimated effect sizes of the instruments on the exposures [30]. Considering that TSMR is challenged by measurement errors, pleiotropy, weak instruments, and selection bias, RAPS proposes an asymptotically averaged estimator by adjusting the contour score to improve robustness and efficiency [33].

#### 2.3.2. Sensitivity Analyses

We calculated the *F* statistics of the selected 303 SNPs to detect the strength of the IVs at a threshold of *F* > 10, which is typically recommended in MR analysis. The *F* statistics was formulated as $F=\frac{R^{2}\left(n\;-\;1\;-\;K\right)}{\left({1\;-\;R}^{2}\right)K}$, where n represents the sample size, *K* represents the number of IVs, and *R*^2^ represents the proportion of variation explained by the SNPs in the exposure [18].

In addition, we assessed the heterogeneities between SNPs using Cochran’s Q-statistics derived from the IVW estimate and the *I*^2^ statistic. The heterogeneity is more likely to be caused by sampling error when the *I*^2^-value is closer to zero and is slight when the *I*^2^-value is less than 0.25 [36].

We performed a leave-one-out analysis as a sensitivity analysis, accompanied with IVW to evaluate the combined effect value of the remaining SNPs. If the combined effect is consistent with the main effect analysis result, no single SNP has an excessive influence on the MR analysis.

All analyses were performed in R software (Version 4.0.5) using the R package (TwoSampleMR, gsmr and simex). Results were presented as the mean effect per 1-SD genetically determined increase in BMI together with the 95% confidence interval (CI); a two-sided *p*-value of less than 0.05 was considered statistically significant.

## 3. Results

### 3.1. Validity of Instrumental Variables

We included 303 SNPs explaining 2.5% ($R^{2}$) of the BMI variation as IVs for BMI-AF causal estimations. The *F*-statistics calculating from the first stage of the MR regression model was 61 [18,37]. The effect estimates of the associations between each SNP and both BMI and AF are reported in the Supplementary Table S1.

### 3.2. Mendelian Randomization

**Figure 1:**
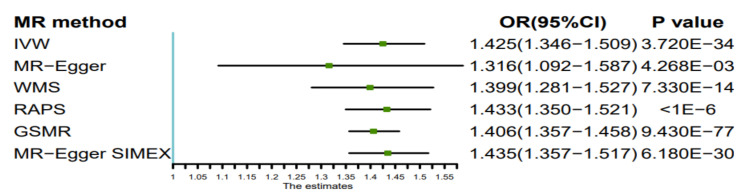
Forest plot of six Mendelian Randomization estimators of the effect of body mass index on atrial fibrillation. Abbreviations: MR, Mendelian randomization; OR, odds ratio; CI, confidence interval; WMS, weighted median estimator; RAPS, the robust adjusted profile score; GSMR, generalized summary data-based Mendelian randomization; SIMEX, simulation extrapolation.

**Figure 2:**
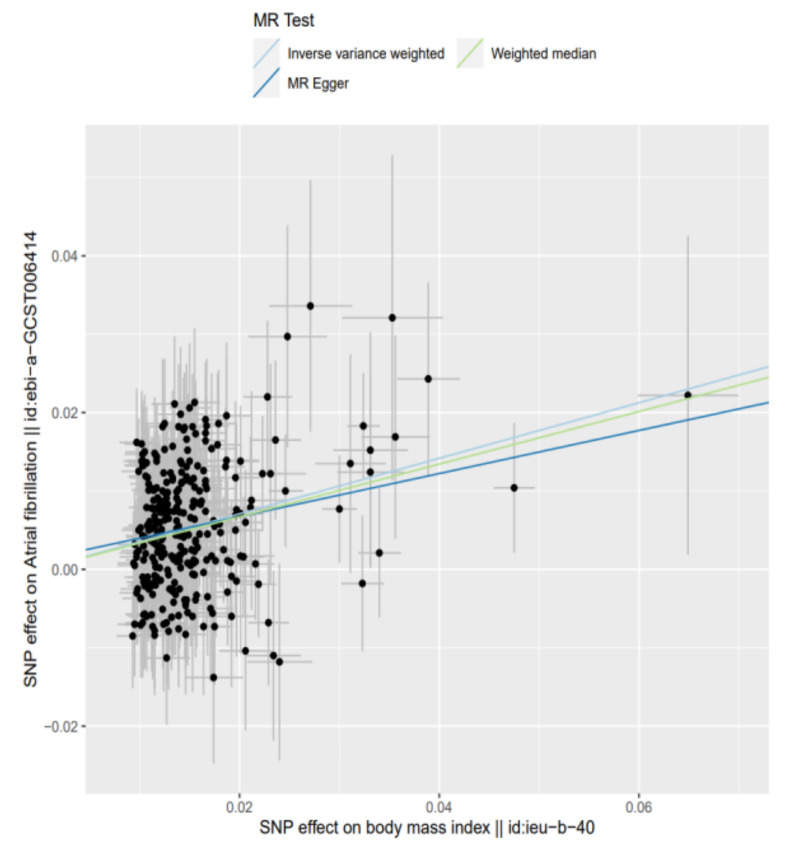
Scatter plot of SNPs associated with BMI and the risk of AF. The plot related the effect sizes of the SNP−BMI association (*x*−axis, SD units) and the SNP−AF associations (*y*−axis, log (OR)) with 95% confidence intervals. The regression slopes of the lines correspond to causal estimates using three Mendelian randomization methods (the inverse variance weighted method, MR-Egger regression, and weighted median estimator). Abbreviations: BMI, body mass index; AF, atrial fibrillation; MR, Mendelian randomization; SNP, single-nucleotide polymorphism.

Table 1 shows the MR estimates of increased BMI with risk of AF. In particular, the results of five methods illustrated that the risk of AF increased with the increment of BMI, and all of them achieved consistently statistical significance. Based on the IVW method, we found a causal relationship between BMI and AF risk; a 1-standard deviation (SD) genetically determined increased BMI was causally associated with an additional 42.5% relative risk of AF (N = 303 SNPs; OR = 1.425; 95% CI = 1.346–1.509; *p* = 3.720 × 10^−^^34^). The estimates from MR-Egger regression, WME, GSMR, and RAPS analyses were consistent with these results (Table 1, Figure 1 and Figure 2).

Since the $I_{GX}^{2}$-statistic of the combined genetic variants for BMI was 0.778, the MR-Egger regression may violate the NOME assumption. Therefore, we performed a SIMEX of the MR-Egger estimate, which improved the estimated value of causal effect with a value of 1.435 closer to the result obtained by the IVW method (Table 1, Figure 1). The test for potential horizontal pleiotropy suggests no significant violation (regression intercepts of 0.0012, 95%CI = −0.0015–0.0040, *p* = 0.380; and −0.0002 95%CI = −0.0010–0.0006, *p* = 0.610), for with or without SIMEX correction, respectively. Neither Cochran’s *Q* test nor *I*^2^-value supported the presence of heterogeneity for the analyses of BMI with AF (*Q*-statistic, 277.28; *p* = 0.84; *I*^2^ = 8.91%) (Table 2).

**Figure 3:**
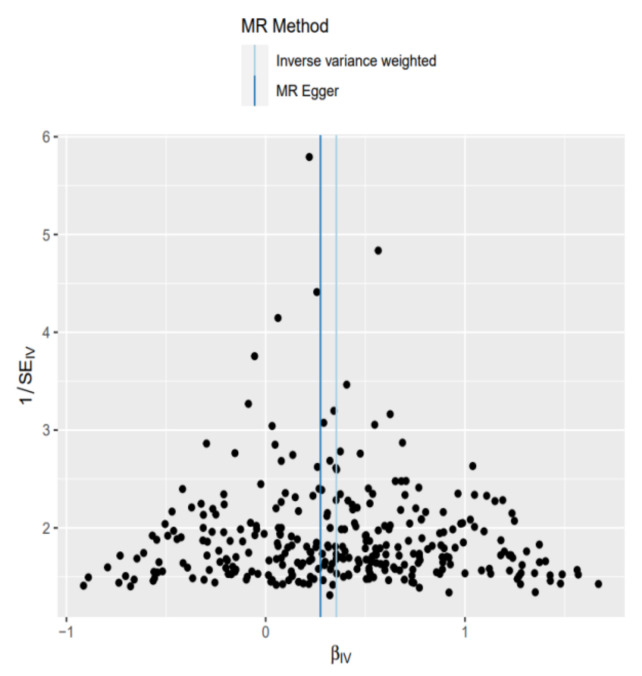
Funnel plot to assess the robustness. Scattering points represented the effect estimated using a single SNP as an instrumental variable. The vertical lines denoted the overall estimate obtained by the inverse variance weighted estimate and the MR-Egger regression.

In addition, there was no single SNP showing a significant impact on the MR estimation results based on leave-one-out analysis, with all significant estimates ranging from 1.42 to 1.43 (Supplementary Table S2). Figure 3 shows the distribution of the increased BMI effect on AF risks was symmetrical when a single SNP was used as an IV.

## 4. Discussion

In this TSMR study, we found a positive causality between the BMI and risk of AF, showing an average of 42.5% increased risk of AF with per 1-SD increment of in BMI. Our findings are in line with previous observational studies [38,39]. Given the random distribution of genotypes in the general population with respect to BMI, as well as the fixed nature of germline genotypes, these results should be less susceptible to confounding and reverse causation than those generated by observational studies.

Although the pathophysiological mechanisms sustaining the effect of BMI on AF have yet to be elucidated entirely, as the surrogate marker of obesity, the findings in previous studies suggest obesity may contribute to this unfortunate outcome through multi-pathways, such as hemodynamic changes, altering epicardial adipose tissue, atrial remodeling, and inflammation [40,41]. 

Excessive adipose accumulation increases total and central blood volume to help perfuse excess tissue, which results in a high cardiac output state and left ventricular (LV) enlargement. In most obese individuals, augmentation of cardiac output predisposes to heart structure remodeling, which lays up the basis for AF [42,43]. In addition, neurohumoral and metabolic disorders caused by obesity, including increased insulin resistance, activation of the renin–angiotensin–aldosterone system, and autonomic dysfunction, also drive cardiac changes in the structure and function [44]. In recent years, the role of epicardial adipose tissue, providing paracrine and autocrine functions in the development of AF, has been recognized [45,46]. The adipokines secreted by epicardial fat may have a significant pro-fibrotic effect on the atrial myocardium and could facilitate atrial myocardial remodeling [46,47]. Other probable mechanisms, such as fat infiltration, inflammation, and oxidative stress, caused by epicardial fat are also implicated in the initiation and maintenance of arrhythmogenesis [45]. Although the mechanism has not been confirmed, studies have shown that the amount of pericardial fat is associated with an increased prevalence of AF [48]. In addition, the systemic pro-inflammatory state characterized by obesity is an essential contributor as inflammation is strongly related to AF [49].

This TSMR analysis had several strengths; (1) Compared with traditional observational studies, the MR method enabled us to provide more reliable effect estimates as it reduced the impact of confounders and reverse causality; (2) the included summary data were based on individuals of European descent, which largely mitigated the effects of population stratification; (3) the identification and selection of the IVs were through a rigorous procedure, which reduced the bias due to the unsuitable IVs.

Our study also had certain limitations: (1) we were unable to perform a stratified analysis upon gender and age due to the lack of individual information in secondary data; (2) the MR method assumes a linear association of exposure-outcome effect; hence the nonlinear relationship between BMI and AF risk was unable to be assessed; (3) the European ancestry of the samples hampered the promotion of our findings to other populations.

## 5. Conclusions

In summary, this TSMR study supports the genetic causality between the increased BMI and AF risks. This finding adds to further evidence that maintaining a healthy BMI is critical for individuals at risk of AF.

## Figures and Tables

**Table 1:** MR estimates from each method of assessing the causal effect of BMI on the risk of AF.

MR Method	No. of SNPs	Beta	SE	P	OR (95%CI)
Inverse variance weighted	303	0.354	0.029	3.720 × 10^−34^	1.425 (1.346–1.509)
MR-Egger	303	0.275	0.095	4.268 × 10^−3^	1.316 (1.092–1.587)
Weighted median estimator	303	0.336	0.045	7.330 × 10^−14^	1.399 (1.281–1.527)
RAPS	303	0.360	0.030	<1 × 10^−6^	1.433 (1.350–1.521)
GSMR	303	0.341	0.018	9.430 × 10^−77^	1.406 (1.357–1.458)
MR-Egger SIMEX	303	0.361	0.028	6.180 × 10^−30^	1.435 (1.357–1.517)

Note: in the RAPS model, the estimated overdispersion parameter was minimal, so the simple model without overdispersion was used. Abbreviations: BMI, body mass index; AF, atrial fibrillation; MR, Mendelian randomization; SNP, single-nucleotide polymorphism; SE, standard error; OR, odds ratio; CI, confidence interval; RAPS, the robust adjusted profile score; GSMR, generalized summary data-based Mendelian randomization; SIMEX, simulation extrapolation.

**Table 2:** The heterogeneity and pleiotropy tests of the instrumental variables.

Cochran’s *Q* Test		*I* ^2^	MR-Egger		MR-Egger SIMEX	
*Q*	*p*		Intercept (95%CI)	*p*	Intercept (95%CI)	*p*
277.28	0.84	8.91%	0.0012(−0.0015–0.0040)	0.38	−0.0002 (−0.0010–0.0006)	0.61

Note: *I*^2^ = (*Q* − *df*)/*Q*. Abbreviations: MR, Mendelian randomization; CI, confidence interval; SIMEX, simulation extrapolation.

## Data Availability

These data were derived from the following resources available in the public websites: the exposure data for BMI https://portals.broadinstitute.org/collaboration/giant/index.php/GIANT_consortium_data_files, and the outcome data for AF http://csg.sph.umich.edu/willer/public/afib2018 (all accessed on 28 April 2021).

## Supplementary Materials

The following supporting information can be downloaded at: https://www.mdpi.com/article/10.3390/nu14091878/s1, Figure S1: Flow chart for quality control of the instrumental variables for MR analysis; Table S1: Characteristics of the SNPs associated with BMI and with AF; Table S2: The combined effect estimators of the remaining 302 SNPs obtained by IVW after removing each SNP.
