# Supplementary material for: Body Mass Index and the Risk of Atrial Fibrillation: A Mendelian Randomization Study
Source: Nutrients. 2022 Apr 29;14(9):1878. doi: 10.3390/nu14091878 (PMC9101688; doi:10.3390/nu14091878)
Supplement: Supplementary file 1 [file nutrients-14-01878-s001.zip › nutrients-1695446-supplementary.pdf]

Supplementary material

Article

Body Mass Index and the Risk of Atrial Fibrillation: A Mendelian Randomization Study

Mi Ma [1], Hong Zhi [2], Sheng-Yi Yang[1], Evan Yi-Wen Yu[1, 3] and Li-Na Wang [1,*]

[1] Key Laboratory of Environmental Medicine Engineering, Ministry of Education, Department of Epidemiology & Biostatistics, School of Public Health, Southeast University, Nanjing, China.
[2] Department of Cardiology, ZhongDa Hospital, Southeast University, Nanjing, China.
[3] CAPHRI Care and Public Health Research Institute, School of Nutrition and Translational Research in Metabolism, Maastricht University, Maastricht, the Netherlands.
* Correspondence: lnwang@seu.edu.cn

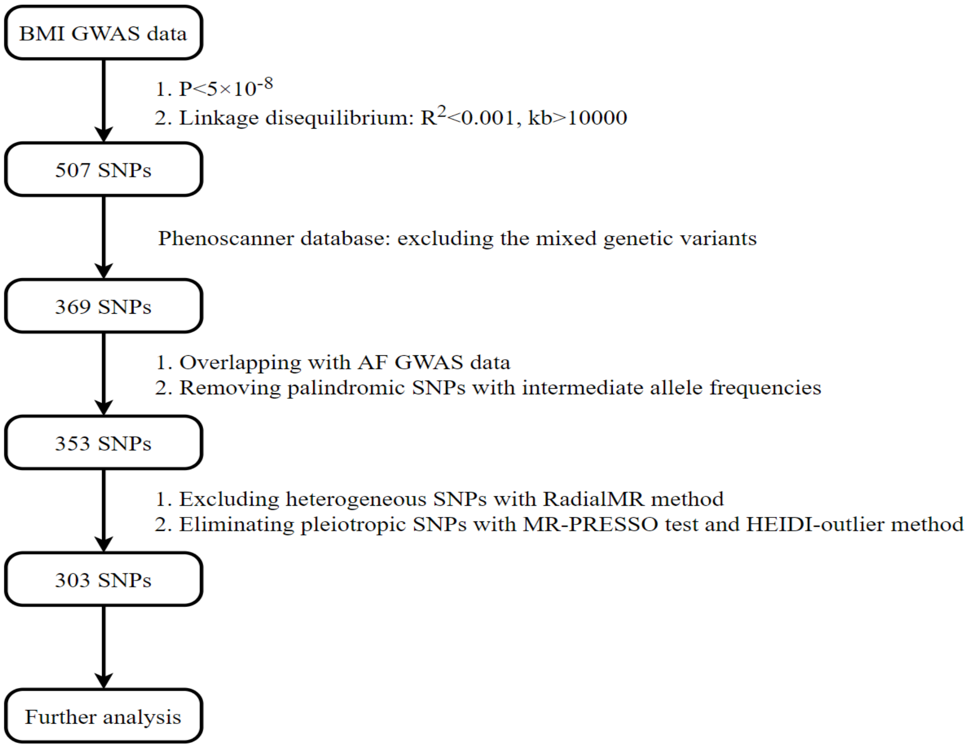

Figure S1. Flow chart for quality control of the instrumental variables for MR analysis

Table S1. Characteristics of the SNPs associated with BMI and with AF

SNP	Chr	Pos	EA	Association with BMI				Association with AF			
				EAF	Beta	SE	P	EAF	Beta	SE	P
rs10009336	4	44480783	T	0.1638	-0.0140	0.0022	2.20E-10	0.1564	-0.0018	0.0090	0.8416
rs1006896	3	88104411	C	0.1061	-0.0234	0.0027	5.50E-18	0.1129	0.0110	0.0108	0.3058
rs10132280	14	25928179	A	0.3017	-0.0223	0.0018	5.60E-35	0.302	-0.0122	0.0073	0.0953
rs10169594	2	41637688	C	0.3596	0.0121	0.0018	2.00E-11	0.3739	0.0073	0.0070	0.2973
rs10197031	2	105454590	C	0.2834	0.0166	0.0019	1.90E-18	0.2918	0.0191	0.0073	0.0092
rs10243319	7	147674678	C	0.3939	-0.0107	0.0018	1.20E-09	0.3967	0.0016	0.0068	0.8125
rs10247983	7	114590228	A	0.9213	0.0201	0.0033	1.70E-09	0.8999	0.0017	0.0124	0.8889
rs10248136	7	39077397	T	0.5142	-0.0097	0.0017	2.00E-08	0.5051	-0.0016	0.0067	0.8043
rs10269783	7	49616203	A	0.3896	0.0133	0.0017	1.40E-15	0.3963	-0.0028	0.0068	0.6755
rs1048932	11	115044850	A	0.4162	-0.0160	0.0017	3.80E-22	0.4282	-0.0045	0.0067	0.5032
rs10742752	11	45438374	C	0.6159	0.0124	0.0017	1.10E-13	0.6144	0.0075	0.0068	0.2726
rs10768994	11	43936945	C	0.4337	-0.0114	0.0017	6.40E-12	0.4422	0.0017	0.0067	0.8037
rs10795422	10	16759312	G	0.6905	0.0139	0.0019	9.30E-14	0.6919	-0.0076	0.0074	0.2995
rs10811871	9	23200766	G	0.3829	-0.0108	0.0018	1.60E-09	0.388	-0.0056	0.0068	0.4146
rs10858334	9	137989785	G	0.1415	0.0143	0.0026	2.70E-08	0.1427	0.0046	0.0109	0.6749
rs10867256	9	81367391	T	0.553	-0.0118	0.0017	8.70E-12	0.5635	-0.0047	0.0067	0.4820
rs10914462	1	32125943	G	0.4255	-0.0112	0.0017	1.50E-10	0.4206	-0.0052	0.0067	0.4398
rs10920678	1	190239907	G	0.5709	-0.0155	0.0016	1.50E-21	0.5735	-0.0023	0.0067	0.7331
rs10938397	4	45182527	G	0.4317	0.0324	0.0016	3.40E-86	0.4234	0.0183	0.0067	0.0066
rs10942267	5	80841914	G	0.3088	-0.0156	0.0019	3.90E-17	0.297	0.0039	0.0073	0.5899
rs10953740	7	113460282	G	0.5534	-0.0153	0.0017	1.00E-18	0.5371	-0.0182	0.0067	0.0070
rs10968114	9	27800007	C	0.4681	-0.0113	0.0017	6.10E-11	0.4726	0.0073	0.0067	0.2764
rs10984756	9	122651784	G	0.1048	0.0174	0.0029	1.10E-09	0.1074	-0.0138	0.0109	0.2058
rs11030618	11	29243293	T	0.5679	0.0110	0.0017	2.40E-10	0.5577	0.0061	0.0067	0.3590
rs11105839	12	91237920	A	0.3799	-0.0109	0.0017	1.10E-10	0.3761	-0.0050	0.0069	0.4693
rs11118308	1	219633869	G	0.4703	-0.0101	0.0016	4.80E-10	0.4559	0.0071	0.0067	0.2874
rs1112613	13	53651850	A	0.1762	-0.0133	0.0023	3.40E-09	0.1838	-0.0114	0.0086	0.1861
rs11150911	18	73498528	C	0.7191	-0.0133	0.0018	4.70E-13	0.6953	-0.0117	0.0073	0.1102
rs11165643	1	96924097	T	0.5828	0.0206	0.0017	1.40E-35	0.5854	0.0060	0.0067	0.3755
rs11170468	12	39430048	C	0.2326	-0.0123	0.0019	1.90E-10	0.241	-0.0040	0.0078	0.6088
rs11173522	12	60953472	A	0.2078	0.0128	0.0021	1.10E-09	0.2103	0.0083	0.0081	0.3073
rs11185111	1	107962328	A	0.3042	-0.0129	0.0019	7.70E-12	0.3021	-0.0074	0.0072	0.3039
rs11251352	10	2585792	G	0.5988	0.0109	0.0018	7.00E-10	0.6053	-0.0018	0.0068	0.7943
rs11505821	7	76818677	T	0.0601	0.0311	0.0035	2.70E-19	0.0616	0.0135	0.0139	0.3287
rs11609659	12	108296260	C	0.2371	-0.0154	0.0020	2.20E-14	0.2458	-0.0087	0.0078	0.2664
rs11615578	12	121714935	T	0.2474	0.0130	0.0020	8.10E-11	0.2542	-0.0025	0.0078	0.7446
rs11656076	17	31464270	A	0.2254	-0.0142	0.0021	5.60E-12	0.2258	-0.0135	0.0079	0.0883
rs11672660	19	46180184	T	0.2049	-0.0340	0.0021	1.70E-60	0.2095	-0.0021	0.0082	0.8019
rs11738695	5	108699161	A	0.586	0.0097	0.0017	2.00E-08	0.5843	0.0162	0.0068	0.0165
rs11739877	5	105876806	T	0.6118	0.0117	0.0018	6.60E-11	0.6131	0.0144	0.0068	0.0362
rs11855853	15	78012618	T	0.2649	-0.0145	0.0020	2.40E-13	0.26	-0.0023	0.0077	0.7643
rs1187352	9	87293457	C	0.6518	0.0119	0.0018	6.00E-11	0.6456	0.0045	0.0070	0.5171
rs11951673	5	95861012	T	0.3941	-0.0123	0.0017	1.10E-13	0.3877	-0.0043	0.0068	0.5231
rs12033257	1	112318484	G	0.3835	-0.0146	0.0018	2.40E-15	0.3813	-0.0114	0.0070	0.1036
rs12041258	1	195047936	C	0.2287	-0.0146	0.0020	9.50E-13	0.2368	0.0046	0.0078	0.5614
rs1218822	13	28011963	A	0.6663	0.0168	0.0017	1.90E-22	0.6449	0.0045	0.0070	0.5187

rs12334877	8	67194171	A	0.198	-0.0144	0.0022	7.70E-11	0.1981	-0.0041	0.0083	0.6209
rs12364470	11	134601012	G	0.1626	0.0178	0.0022	1.10E-15	0.1676	0.0159	0.0091	0.0798
rs12416812	11	888632	A	0.5088	0.0111	0.0016	6.10E-12	0.5191	-0.0022	0.0066	0.7401
rs1241986	18	6873954	A	0.8479	-0.0139	0.0024	1.10E-08	0.8259	-0.0102	0.0092	0.2701
rs12422552	12	14413931	C	0.2663	-0.0134	0.0020	1.60E-11	0.2567	-0.0013	0.0077	0.8637
rs12546578	8	85085268	A	0.7246	0.0146	0.0020	1.00E-13	0.7239	-0.0083	0.0076	0.2752
rs12564992	1	174478100	G	0.1144	0.0196	0.0026	5.30E-14	0.1082	0.0050	0.0106	0.6373
rs12629015	3	119618053	G	0.1852	-0.0135	0.0023	2.10E-09	0.1761	-0.0211	0.0086	0.0140
rs1266874	6	51779638	G	0.3558	0.0140	0.0018	9.80E-15	0.3362	0.0011	0.0070	0.8781
rs12675063	8	132879047	T	0.1131	0.0156	0.0026	1.30E-09	0.1109	0.0035	0.0104	0.7394
rs1268065	6	126042783	A	0.4794	-0.0102	0.0017	1.00E-09	0.4805	-0.0160	0.0067	0.0167
rs12718572	7	50573325	T	0.4024	-0.0117	0.0018	3.00E-11	0.4022	-0.0138	0.0068	0.0416
rs12762034	10	33969931	C	0.07583	0.0240	0.0032	7.30E-14	0.0771	-0.0118	0.0125	0.3476
rs1285997	14	91513029	G	0.7153	0.0142	0.0019	1.20E-13	0.6976	0.0124	0.0073	0.0914
rs12888545	14	88308044	G	0.2519	0.0136	0.0020	9.10E-12	0.258	-0.0032	0.0077	0.6797
rs12905439	15	99521883	G	0.3393	-0.0118	0.0018	1.40E-10	0.3572	-0.0022	0.0072	0.7607
rs12922346	16	82438337	C	0.2657	0.0136	0.0020	1.00E-11	0.2679	0.0075	0.0076	0.3271
rs12939549	17	78611724	G	0.4335	-0.0180	0.0016	2.70E-28	0.4387	-0.0058	0.0067	0.3844
rs1296328	4	137083193	C	0.5657	-0.0179	0.0018	4.90E-24	0.5568	-0.0186	0.0068	0.0063
rs13047416	21	40309436	G	0.3769	-0.0154	0.0018	2.20E-17	0.3776	-0.0008	0.0070	0.9075
rs13069244	3	180441172	A	0.07747	0.0187	0.0032	3.00E-09	0.0766	0.0139	0.0130	0.2834
rs13110266	4	162129844	A	0.4065	-0.0117	0.0017	1.90E-12	0.4147	-0.0143	0.0068	0.0341
rs13147390	4	80712000	C	0.3569	0.0103	0.0018	1.00E-08	0.3594	-0.0010	0.0070	0.8909
rs13174863	5	139080745	G	0.1548	0.0192	0.0023	2.90E-16	0.1501	-0.0009	0.0095	0.9249
rs13191362	6	163033350	G	0.1198	-0.0236	0.0025	5.90E-21	0.1292	-0.0165	0.0101	0.1022
rs1320903	3	131758077	A	0.3174	0.0216	0.0018	9.20E-32	0.3127	0.0007	0.0071	0.9171
rs1321432	20	6614691	C	0.6321	0.0201	0.0018	3.50E-29	0.6287	0.0138	0.0070	0.0480
rs13250058	8	112270826	T	0.6771	0.0112	0.0018	2.90E-10	0.6792	-0.0058	0.0072	0.4200
rs13263601	8	14095900	C	0.3478	0.0154	0.0018	2.20E-17	0.3415	0.0116	0.0070	0.0993
rs1327259	6	51177811	G	0.3872	-0.0155	0.0018	1.70E-18	0.3828	-0.0064	0.0068	0.3462
rs13287131	9	92119579	C	0.2491	0.0123	0.0020	6.80E-10	0.2581	0.0079	0.0077	0.3005
rs1330052	13	86536006	G	0.3504	0.0132	0.0018	1.50E-13	0.3667	0.0017	0.0069	0.8107
rs1365466	18	36182440	T	0.7406	-0.0137	0.0019	3.30E-13	0.7327	-0.0091	0.0076	0.2283
rs1371108	2	81816251	A	0.3247	0.0119	0.0018	9.00E-11	0.3186	0.0060	0.0071	0.3978
rs1412235	9	28410996	C	0.3175	0.0246	0.0017	6.00E-45	0.3177	0.0100	0.0071	0.1618
rs1430387	18	58227112	C	0.4295	-0.0114	0.0017	5.80E-11	0.4314	-0.0101	0.0067	0.1309
rs1431659	8	73439070	G	0.7344	-0.0196	0.0019	6.00E-24	0.7184	-0.0069	0.0075	0.3536
rs1445652	2	155668460	A	0.1855	0.0123	0.0022	4.30E-08	0.1878	0.0182	0.0086	0.0341
rs1465900	11	76473138	C	0.2188	-0.0125	0.0020	4.80E-10	0.2186	-0.0153	0.0080	0.0570
rs1476322	3	161446055	A	0.569	0.0101	0.0017	5.00E-09	0.5544	0.0053	0.0066	0.4249
rs1477199	16	53712135	G	0.1451	0.0228	0.0024	9.40E-22	0.1392	0.0220	0.0097	0.0238
rs1492767	4	55221467	T	0.4957	0.0094	0.0016	1.00E-08	0.5184	0.0008	0.0066	0.8993
rs1503526	5	63020706	C	0.4838	0.0140	0.0017	5.50E-17	0.4878	0.0043	0.0066	0.5177
rs1522569	4	171632637	G	0.1819	-0.0164	0.0022	2.90E-13	0.1729	0.0073	0.0087	0.4045
rs1528435	2	181550962	T	0.6331	0.0164	0.0017	9.10E-23	0.6191	0.0126	0.0068	0.0643
rs1535660	9	10371073	C	0.8554	-0.0147	0.0025	5.20E-09	0.837	-0.0166	0.0096	0.0823
rs1538247	6	153395344	C	0.3181	0.0108	0.0019	1.00E-08	0.3116	-0.0010	0.0072	0.8884
rs156201	6	104847441	C	0.7606	0.0123	0.0020	5.80E-10	0.7216	-0.0051	0.0075	0.5022
rs1624134	10	34834482	C	0.4068	0.0101	0.0018	1.10E-08	0.4072	-0.0037	0.0068	0.5810

rs1656377	3	158285280	C	0.5885	0.0099	0.0017	1.60E-08	0.5844	0.0125	0.0067	0.0630
rs1681740	10	118564313	C	0.3933	-0.0115	0.0018	1.10E-10	0.4049	-0.0009	0.0068	0.8938
rs16871902	5	3488462	A	0.4877	0.0125	0.0017	4.60E-13	0.4778	0.0063	0.0067	0.3520
rs17014375	1	209543560	G	0.1348	0.0172	0.0025	1.10E-11	0.1384	0.0154	0.0096	0.1106
rs17033117	3	35443653	T	0.1872	0.0137	0.0022	8.90E-10	0.1878	0.0079	0.0084	0.3470
rs17113297	10	102395982	T	0.2082	0.0166	0.0021	2.10E-15	0.2103	0.0164	0.0081	0.0438
rs17119937	8	14502274	C	0.06905	0.0212	0.0036	5.60E-09	0.0679	0.0088	0.0139	0.5250
rs17203016	2	208255518	G	0.196	0.0150	0.0020	2.10E-13	0.2089	0.0206	0.0082	0.0121
rs17238110	15	62150364	G	0.1634	-0.0353	0.0050	2.00E-12	0.023	-0.0321	0.0207	0.1216
rs17311369	15	47709199	T	0.3278	-0.0104	0.0019	3.10E-08	0.3333	-0.0030	0.0071	0.6711
rs17399237	2	35471626	C	0.5497	-0.0129	0.0017	6.70E-14	0.5494	-0.0065	0.0067	0.3276
rs17424296	5	60838903	A	0.3659	-0.0108	0.0018	2.40E-09	0.3648	-0.0117	0.0069	0.0919
rs17425707	1	57874879	C	0.1003	0.0167	0.0028	4.40E-09	0.0972	0.0103	0.0114	0.3628
rs17446257	13	40749213	A	0.1292	0.0153	0.0026	2.90E-09	0.1323	-0.0006	0.0100	0.9514
rs17499593	2	172649755	G	0.1897	0.0125	0.0022	1.10E-08	0.202	0.0087	0.0085	0.3046
rs175165	22	20116015	G	0.3941	-0.0103	0.0018	5.20E-09	0.4072	-0.0147	0.0068	0.0314
rs17535749	3	10027724	A	0.1023	0.0150	0.0027	2.50E-08	0.1089	0.0138	0.0112	0.2163
rs17551974	2	142293146	A	0.1782	-0.0141	0.0022	1.90E-10	0.1903	-0.0198	0.0085	0.0189
rs17636031	10	126594078	C	0.2701	0.0160	0.0019	1.20E-17	0.2785	0.0100	0.0079	0.2098
rs17663412	5	167595121	A	0.1139	0.0157	0.0027	6.10E-09	0.1131	0.0085	0.0105	0.4208
rs17710386	18	63461201	C	0.3319	0.0126	0.0018	1.00E-12	0.3236	0.0070	0.0073	0.3374
rs1830074	7	6718674	C	0.288	0.0115	0.0019	1.40E-09	0.2967	-0.0008	0.0074	0.9137
rs1836303	15	46539116	G	0.3873	0.0116	0.0018	5.30E-11	0.3926	0.0062	0.0068	0.3659
rs1843328	12	17111188	A	0.5085	-0.0099	0.0017	7.90E-09	0.5063	-0.0050	0.0067	0.4569
rs1863652	4	95991417	A	0.3449	-0.0115	0.0018	1.40E-10	0.3477	-0.0054	0.0070	0.4340
rs1884389	20	1410582	T	0.4289	-0.0103	0.0017	4.00E-09	0.4344	-0.0043	0.0068	0.5258
rs1885728	6	5977833	A	0.6787	0.0108	0.0019	1.00E-08	0.6733	0.0056	0.0073	0.4436
rs1891216	1	7728391	G	0.3759	0.0107	0.0018	2.40E-09	0.3785	-0.0060	0.0069	0.3831
rs1896767	16	62838304	A	0.5376	-0.0109	0.0017	2.40E-10	0.556	-0.0101	0.0067	0.1323
rs1927790	13	96922191	C	0.4109	0.0148	0.0016	1.80E-19	0.3989	-0.0055	0.0067	0.4186
rs1937683	10	53679060	T	0.6699	0.0109	0.0018	3.20E-09	0.6656	0.0109	0.0071	0.1219
rs1948080	9	11852043	G	0.3749	-0.0137	0.0018	1.10E-14	0.371	-0.0052	0.0069	0.4488
rs1982441	8	28021769	T	0.1381	0.0175	0.0026	7.00E-12	0.1385	0.0055	0.0097	0.5738
rs1982725	19	30618771	T	0.4778	0.0097	0.0017	3.30E-08	0.4777	0.0021	0.0068	0.7521
rs2007231	1	115266306	T	0.6387	-0.0104	0.0018	5.20E-09	0.6339	0.0058	0.0070	0.4066
rs200810	6	97922184	C	0.3716	-0.0136	0.0017	5.50E-16	0.3837	-0.0044	0.0068	0.5174
rs2009416	5	92415111	T	0.361	-0.0121	0.0018	1.10E-11	0.3577	-0.0027	0.0069	0.6960
rs2051559	4	3298800	C	0.1308	0.0176	0.0026	5.00E-12	0.1376	0.0011	0.0097	0.9070
rs208015	17	46252346	C	0.9216	-0.0356	0.0034	1.40E-25	0.9018	-0.0169	0.0129	0.1899
rs2124499	3	123093541	C	0.3718	-0.0123	0.0017	3.40E-13	0.3792	-0.0100	0.0069	0.1444
rs2143253	20	41987392	A	0.1189	-0.0188	0.0026	1.10E-12	0.1202	-0.0011	0.0102	0.9106
rs2162524	2	230817437	C	0.3321	0.0155	0.0018	4.10E-17	0.3242	0.0105	0.0071	0.1394
rs217671	14	62360464	G	0.2719	0.0144	0.0019	1.30E-13	0.2612	0.0072	0.0075	0.3397
rs2228213	6	12124855	A	0.3481	-0.0139	0.0017	4.60E-16	0.3381	-0.0086	0.0070	0.2174
rs2246012	6	131898208	C	0.1628	0.0158	0.0022	3.10E-13	0.1746	0.0132	0.0087	0.1301
rs2283093	7	126721231	T	0.2066	0.0127	0.0021	3.10E-09	0.2242	0.0076	0.0081	0.3465
rs2306537	12	133423695	G	0.3092	0.0133	0.0019	8.70E-13	0.3269	0.0157	0.0071	0.0278
rs2317299	2	236903093	C	0.5597	-0.0106	0.0017	1.30E-09	0.5439	-0.0119	0.0067	0.0777
rs2357760	6	120213880	A	0.6754	0.0145	0.0017	6.80E-17	0.6498	0.0181	0.0070	0.0097

rs2361988	16	398151	C	0.2539	-0.0155	0.0020	5.20E-15	0.2414	0.0007	0.0078	0.9263
rs2365389	3	61236462	T	0.4143	-0.0174	0.0017	1.30E-25	0.4277	-0.0062	0.0067	0.3581
rs2367112	5	64168193	G	0.4919	-0.0119	0.0016	2.30E-13	0.5014	-0.0030	0.0066	0.6542
rs2423668	20	12430673	C	0.5505	-0.0105	0.0019	2.80E-08	0.546	0.0057	0.0068	0.4036
rs2425840	20	44904838	C	0.4059	0.0119	0.0018	1.60E-11	0.4075	0.0027	0.0068	0.6916
rs2429150	12	2152655	C	0.4164	0.0111	0.0018	2.70E-10	0.4144	0.0037	0.0068	0.5836
rs2479958	13	111984244	G	0.5075	-0.0154	0.0018	1.50E-17	0.5036	-0.0012	0.0068	0.8622
rs2481665	1	62594677	C	0.4408	-0.0161	0.0016	7.20E-23	0.4465	-0.0083	0.0067	0.2125
rs2543132	8	15536311	C	0.8134	0.0146	0.0022	5.00E-11	0.8001	-0.0043	0.0085	0.6180
rs2605603	11	93221105	A	0.4887	-0.0103	0.0016	2.50E-10	0.4755	-0.0139	0.0067	0.0368
rs2608703	12	41846769	A	0.4546	0.0142	0.0017	1.90E-16	0.4642	0.0044	0.0066	0.5031
rs262130	6	142853486	T	0.1969	0.0127	0.0023	1.80E-08	0.1867	-0.0113	0.0085	0.1841
rs2693826	2	6160943	A	0.4421	-0.0137	0.0017	2.00E-15	0.4351	-0.0099	0.0067	0.1387
rs273504	19	18215247	G	0.4266	0.0153	0.0018	4.40E-18	0.4234	0.0080	0.0068	0.2395
rs2791653	1	11129848	G	0.7577	-0.0141	0.0019	1.30E-13	0.7516	-0.0029	0.0078	0.7121
rs2832283	21	30690558	A	0.2208	0.0115	0.0020	5.80E-09	0.2179	-0.0078	0.0082	0.3402
rs2861683	2	67836507	C	0.407	-0.0144	0.0017	1.30E-16	0.3976	-0.0178	0.0067	0.0084
rs287104	19	34290995	A	0.6604	0.0115	0.0017	4.40E-11	0.6593	0.0068	0.0071	0.3425
rs2875762	6	124925032	C	0.2473	0.0139	0.0020	1.20E-11	0.2343	0.0182	0.0079	0.0208
rs2931434	5	73159098	T	0.3168	-0.0104	0.0018	1.40E-08	0.3219	-0.0012	0.0071	0.8703
rs2943465	12	19265921	C	0.9444	0.0248	0.0039	2.00E-10	0.9182	0.0297	0.0141	0.0349
rs294704	5	152519088	T	0.7239	-0.0113	0.0019	4.00E-09	0.7136	-0.0015	0.0075	0.8420
rs3007105	14	47367616	T	0.4697	0.0142	0.0017	1.10E-17	0.4077	0.0111	0.0068	0.0995
rs33500	3	42427191	T	0.8082	-0.0167	0.0022	4.30E-14	0.7921	-0.0183	0.0085	0.0310
rs339991	15	60913637	G	0.5631	0.0124	0.0018	1.20E-12	0.5746	0.0123	0.0067	0.0652
rs349088	11	84814393	A	0.4976	-0.0128	0.0017	1.80E-13	0.4936	0.0001	0.0066	0.9905
rs3731695	2	203820275	C	0.5582	0.0116	0.0016	7.90E-13	0.5453	0.0041	0.0067	0.5365
rs3732084	2	207174316	C	0.6139	0.0107	0.0018	1.10E-09	0.6084	0.0079	0.0068	0.2432
rs3736485	15	51748610	G	0.5443	-0.0134	0.0016	2.50E-16	0.5563	0.0042	0.0067	0.5271
rs3749897	6	42532102	T	0.4172	0.0122	0.0018	8.40E-12	0.4038	0.0038	0.0067	0.5697
rs3764835	2	159519368	A	0.1528	-0.0141	0.0024	3.10E-09	0.1546	-0.0002	0.0093	0.9844
rs3772882	3	81808602	A	0.3661	0.0127	0.0018	6.60E-13	0.3853	0.0091	0.0068	0.1819
rs3800229	6	108996963	T	0.7123	0.0175	0.0018	1.40E-22	0.707	-0.0073	0.0073	0.3204
rs3800637	7	137403432	C	0.336	0.0115	0.0018	5.10E-10	0.3391	0.0148	0.0071	0.0356
rs3806114	6	20482335	A	0.6773	-0.0113	0.0018	3.40E-10	0.6592	0.0032	0.0072	0.6575
rs3806572	2	55238677	A	0.2788	-0.0145	0.0019	1.60E-14	0.2834	0.0039	0.0074	0.5981
rs3807645	7	77830091	A	0.221	-0.0166	0.0021	2.40E-15	0.2209	-0.0074	0.0081	0.3554
rs380857	9	101491066	A	0.8878	-0.0151	0.0027	3.60E-08	0.8621	-0.0005	0.0103	0.9647
rs38314	7	70067315	A	0.4912	-0.0120	0.0017	4.70E-12	0.492	-0.0060	0.0067	0.3656
rs3844598	5	140992235	G	0.521	0.0095	0.0017	3.80E-08	0.5357	-0.0070	0.0066	0.2894
rs3902951	14	69789755	G	0.2455	0.0134	0.0020	7.00E-12	0.255	0.0074	0.0077	0.3410
rs3904244	10	27361527	A	0.1377	0.0155	0.0025	4.30E-10	0.1391	0.0213	0.0094	0.0236
rs3935648	17	79085335	G	0.2328	-0.0125	0.0022	6.80E-09	0.2177	-0.0186	0.0082	0.0242
rs3977755	10	104420210	T	0.2804	-0.0135	0.0019	5.90E-13	0.2639	-0.0015	0.0075	0.8419
rs4072917	8	143300279	A	0.4694	0.0115	0.0018	6.90E-11	0.4765	0.0077	0.0067	0.2531
rs4307239	7	24354300	G	0.4578	0.0115	0.0017	3.90E-11	0.4544	-0.0084	0.0067	0.2115
rs4310573	11	97855562	T	0.7813	0.0116	0.0021	3.50E-08	0.7611	0.0003	0.0080	0.9747
rs4358081	2	29100642	C	0.4631	0.0097	0.0017	1.50E-08	0.4707	-0.0030	0.0066	0.6547
rs4430672	14	63094407	C	0.8004	-0.0127	0.0022	3.90E-09	0.7861	-0.0111	0.0083	0.1818

rs4482463	2	205375909	A	0.9213	-0.0331	0.0033	2.80E-23	0.895	-0.0124	0.0119	0.2976
rs4518345	5	27185904	A	0.2842	-0.0117	0.0019	1.00E-09	0.2837	-0.0031	0.0074	0.6723
rs4556997	2	100814858	A	0.1349	0.0197	0.0024	6.90E-17	0.137	-0.0015	0.0096	0.8740
rs4589691	2	144051398	G	0.1579	0.0141	0.0024	4.70E-09	0.152	0.0019	0.0093	0.8349
rs4639527	2	416815	G	0.3012	0.0172	0.0019	3.30E-20	0.3102	0.0017	0.0073	0.8155
rs4653017	1	33776728	T	0.6818	0.0122	0.0018	4.50E-11	0.6748	0.0051	0.0072	0.4774
rs4671328	2	58935282	G	0.5533	-0.0219	0.0017	2.20E-36	0.526	0.0019	0.0067	0.7795
rs4722398	7	3125220	T	0.1336	0.0158	0.0025	3.60E-10	0.134	0.0028	0.0098	0.7789
rs4783830	16	54255346	A	0.3074	-0.0105	0.0019	2.40E-08	0.3055	-0.0150	0.0072	0.0368
rs4786903	16	6697104	G	0.7368	0.0125	0.0020	3.50E-10	0.7117	0.0020	0.0076	0.7939
rs4800191	18	22461398	C	0.6369	0.0103	0.0017	2.50E-09	0.6326	-0.0068	0.0070	0.3316
rs4818225	21	42629895	G	0.6606	0.0117	0.0018	2.30E-10	0.6502	0.0104	0.0071	0.1424
rs4820408	22	40604945	G	0.592	-0.0151	0.0017	2.10E-19	0.6048	-0.0066	0.0069	0.3344
rs4858193	3	20441050	C	0.2779	-0.0129	0.0019	1.60E-11	0.2988	0.0079	0.0074	0.2856
rs4864201	4	130731284	C	0.6469	-0.0141	0.0017	1.50E-16	0.6439	-0.0138	0.0069	0.0469
rs4880341	10	133992689	T	0.5606	-0.0118	0.0017	1.10E-11	0.5763	-0.0091	0.0068	0.1808
rs4906908	15	27040082	G	0.5253	0.0103	0.0017	2.50E-09	0.5219	0.0132	0.0067	0.0471
rs491711	11	28742220	C	0.316	-0.0115	0.0019	1.10E-09	0.3115	-0.0102	0.0073	0.1623
rs4929923	11	8639200	C	0.6376	0.0181	0.0017	7.20E-27	0.6387	0.0047	0.0069	0.4919
rs4936175	11	132641959	C	0.4445	0.0122	0.0017	1.40E-12	0.4541	0.0016	0.0067	0.8062
rs4954638	2	137435455	C	0.2492	-0.0118	0.0020	2.90E-09	0.2351	-0.0042	0.0077	0.5903
rs4986044	17	21261560	T	0.4687	-0.0164	0.0016	3.30E-23	0.4533	0.0004	0.0067	0.9548
rs538579	3	62711674	C	0.3228	0.0137	0.0019	1.30E-13	0.3264	0.0010	0.0071	0.8920
rs543874	1	177889480	G	0.1952	0.0475	0.0020	1.2E-122	0.207	0.0104	0.0082	0.2052
rs559231	18	39644247	T	0.3956	0.0135	0.0018	2.40E-14	0.3949	-0.0017	0.0068	0.8010
rs577525	10	99769388	C	0.5676	0.0166	0.0017	9.70E-22	0.5688	0.0113	0.0067	0.0916
rs592483	11	69445173	T	0.5716	-0.0147	0.0017	2.00E-18	0.5654	-0.0131	0.0068	0.0518
rs6443750	3	181329682	C	0.8068	0.0148	0.0021	3.20E-12	0.7872	0.0008	0.0088	0.9306
rs6448587	4	28561990	C	0.1891	-0.0167	0.0023	2.30E-13	0.1765	-0.0012	0.0086	0.8860
rs6512302	20	62691550	C	0.7511	0.0142	0.0020	2.10E-12	0.7259	0.0110	0.0085	0.1955
rs6545714	2	59307725	A	0.6139	-0.0191	0.0017	9.10E-31	0.6041	-0.0009	0.0067	0.8988
rs6561943	13	58356761	T	0.2595	0.0119	0.0019	4.20E-10	0.2637	0.0080	0.0076	0.2867
rs657452	1	49589847	G	0.6216	-0.0188	0.0017	7.20E-29	0.6038	0.0029	0.0068	0.6664
rs6587552	1	151018861	G	0.7591	-0.0173	0.0020	1.60E-17	0.738	0.0056	0.0077	0.4650
rs6591407	11	56914157	A	0.1861	-0.0118	0.0021	1.90E-08	0.1853	-0.0091	0.0085	0.2850
rs6593688	1	96322205	G	0.3733	0.0137	0.0018	8.60E-15	0.381	0.0062	0.0068	0.3655
rs6692586	1	23299906	G	0.832	-0.0192	0.0023	1.10E-16	0.8191	0.0060	0.0090	0.5070
rs6764533	3	196088464	A	0.359	0.0116	0.0018	1.40E-10	0.3667	0.0041	0.0069	0.5513
rs6772756	3	182312152	G	0.3372	-0.0104	0.0019	4.00E-08	0.3349	-0.0133	0.0073	0.0703
rs6785245	3	82647990	C	0.3969	0.0132	0.0017	4.00E-14	0.4066	-0.0061	0.0067	0.3686
rs6804842	3	25106437	G	0.572	0.0156	0.0017	3.60E-21	0.5751	0.0173	0.0067	0.0101
rs6841761	4	25423538	T	0.5252	-0.0131	0.0016	6.40E-16	0.5323	-0.0123	0.0066	0.0636
rs685870	11	64111928	C	0.7035	0.0120	0.0019	2.40E-10	0.6913	0.0002	0.0072	0.9807
rs7025938	9	103088321	G	0.3187	0.0166	0.0019	3.70E-19	0.3135	0.0174	0.0071	0.0142
rs7037266	9	6942940	A	0.3739	-0.0112	0.0018	3.50E-10	0.3553	-0.0078	0.0069	0.2602
rs705217	1	34581472	G	0.3652	-0.0102	0.0018	9.30E-09	0.3644	-0.0138	0.0076	0.0683
rs7138803	12	50247468	A	0.3772	0.0300	0.0017	2.30E-71	0.375	0.0077	0.0068	0.2618
rs7148846	14	40133821	G	0.1896	0.0124	0.0022	2.20E-08	0.1839	-0.0070	0.0085	0.4089
rs7172627	15	31877690	G	0.4719	0.0117	0.0017	1.10E-11	0.484	-0.0010	0.0067	0.8857

rs7196720	16	24534662	C	0.5068	-0.0129	0.0017	7.30E-14	0.5234	0.0007	0.0067	0.9113
rs7334078	13	99120484	C	0.2882	-0.0121	0.0019	2.20E-10	0.279	-0.0073	0.0074	0.3240
rs7488867	12	103699685	T	0.2639	-0.0204	0.0020	8.40E-24	0.2522	-0.0016	0.0076	0.8328
rs7519259	1	66434743	A	0.5356	0.0125	0.0017	3.80E-13	0.5313	0.0098	0.0066	0.1386
rs7535528	1	2444414	A	0.3741	-0.0152	0.0018	1.40E-16	0.3594	-0.0025	0.0070	0.7169
rs7550711	1	110082886	T	0.03058	0.0649	0.0050	3.20E-38	0.0289	0.0222	0.0203	0.2733
rs7551507	1	74995225	T	0.5633	-0.0184	0.0016	9.30E-30	0.5565	-0.0025	0.0067	0.7130
rs7599312	2	213413231	A	0.2652	-0.0186	0.0019	6.90E-24	0.2749	-0.0131	0.0075	0.0799
rs7615297	3	156299313	G	0.1465	-0.0149	0.0024	5.70E-10	0.1538	-0.0044	0.0092	0.6309
rs7637852	3	44041777	G	0.6951	-0.0139	0.0019	1.70E-13	0.6831	0.0059	0.0073	0.4131
rs765875	6	143185683	T	0.4808	-0.0121	0.0017	3.00E-12	0.4849	0.0014	0.0065	0.8283
rs7683836	4	180167906	A	0.5405	-0.0114	0.0017	6.30E-11	0.5415	-0.0088	0.0067	0.1897
rs768840	14	73143457	A	0.4183	0.0114	0.0018	2.00E-10	0.4314	0.0034	0.0069	0.6256
rs7694732	4	115124089	G	0.4378	-0.0099	0.0017	8.70E-09	0.4275	-0.0024	0.0067	0.7232
rs7704281	5	50591460	A	0.04531	0.0271	0.0041	6.50E-11	0.0471	0.0336	0.0160	0.0352
rs7715256	5	153537893	T	0.5781	-0.0166	0.0016	2.20E-24	0.5789	-0.0108	0.0067	0.1073
rs7730004	5	43191033	T	0.6693	0.0148	0.0018	9.10E-16	0.6601	0.0152	0.0071	0.0311
rs7730898	5	170459675	A	0.729	0.0168	0.0018	4.50E-20	0.7141	-0.0035	0.0075	0.6409
rs774246	7	26990816	G	0.1444	0.0153	0.0025	5.40E-10	0.141	-0.0060	0.0096	0.5342
rs7780752	7	93241640	C	0.36	0.0139	0.0018	1.00E-14	0.3602	0.0055	0.0070	0.4332
rs7788008	7	112972483	A	0.4445	-0.0157	0.0017	1.10E-19	0.4339	0.0033	0.0067	0.6164
rs7811342	7	138794618	C	0.1058	-0.0197	0.0029	1.10E-11	0.0991	-0.0076	0.0109	0.4863
rs7819514	8	93204442	A	0.3216	-0.0107	0.0018	5.70E-09	0.3333	-0.0136	0.0071	0.0548
rs7826312	8	32400115	C	0.5879	0.0104	0.0017	4.90E-10	0.5842	-0.0016	0.0067	0.8108
rs7844647	8	34503776	C	0.2681	-0.0123	0.0018	2.80E-11	0.2724	-0.0027	0.0074	0.7188
rs7869771	9	94180627	C	0.2647	-0.0140	0.0019	4.90E-13	0.2679	0.0025	0.0075	0.7421
rs7871866	9	131027982	C	0.1531	0.0187	0.0024	2.30E-14	0.1542	0.0196	0.0093	0.0346
rs7899106	10	87410904	G	0.04777	0.0331	0.0037	1.00E-18	0.0555	0.0152	0.0150	0.3098
rs7925214	11	130794253	T	0.5133	0.0147	0.0018	4.40E-17	0.5325	0.0118	0.0068	0.0819
rs7998796	13	81020036	G	0.3373	0.0105	0.0018	1.10E-08	0.334	0.0041	0.0070	0.5590
rs8027205	15	98280959	G	0.3967	-0.0108	0.0018	1.40E-09	0.3821	-0.0056	0.0069	0.4126
rs8036040	15	36402716	A	0.4932	0.0109	0.0017	2.70E-10	0.4981	-0.0025	0.0066	0.7103
rs806600	5	172914939	G	0.475	-0.0095	0.0017	3.30E-08	0.4634	-0.0005	0.0067	0.9350
rs8071182	17	55336155	A	0.1735	0.0133	0.0022	2.10E-09	0.1863	-0.0025	0.0087	0.7742
rs8090983	18	52586691	G	0.3314	0.0118	0.0018	2.00E-10	0.338	0.0046	0.0070	0.5122
rs8097783	18	58051294	A	0.07554	-0.0389	0.0031	7.20E-36	0.0761	-0.0243	0.0123	0.0486
rs8123881	20	15819495	G	0.1299	0.0196	0.0024	4.40E-16	0.1376	0.0117	0.0097	0.2237
rs8181823	13	65477940	C	0.7614	0.0127	0.0020	4.10E-10	0.74	-0.0068	0.0077	0.3816
rs818524	1	85201228	C	0.6939	0.0106	0.0019	3.40E-08	0.693	0.0020	0.0074	0.7816
rs825688	16	73595718	T	0.456	-0.0095	0.0017	4.70E-08	0.4492	-0.0032	0.0067	0.6351
rs845084	10	125220036	A	0.2678	0.0140	0.0020	1.30E-12	0.2663	0.0073	0.0075	0.3274
rs852056	20	17102860	C	0.7584	-0.0128	0.0020	1.80E-10	0.7493	-0.0098	0.0078	0.2135
rs872281	14	40834177	T	0.1728	-0.0151	0.0023	4.70E-11	0.1758	-0.0112	0.0087	0.2005
rs895330	19	4060707	G	0.1924	-0.0201	0.0023	5.50E-19	0.1922	-0.0071	0.0088	0.4173
rs9294260	6	83433228	A	0.4731	0.0147	0.0016	1.80E-19	0.4745	-0.0039	0.0067	0.5588
rs930295	2	50233352	C	0.8417	-0.0211	0.0023	1.00E-19	0.812	-0.0079	0.0090	0.3771
rs9304665	19	47602577	A	0.7633	0.0229	0.0020	2.90E-29	0.7481	-0.0068	0.0080	0.3983
rs9362662	6	90296588	G	0.5201	-0.0112	0.0017	1.20E-10	0.5091	-0.0103	0.0066	0.1190
rs9367368	6	13189275	C	0.3033	-0.0121	0.0018	1.00E-11	0.3065	-0.0028	0.0072	0.6987

rs9408882	9	118664402	A	0.4594	-0.0093	0.0016	1.30E-08	0.4629	0.0085	0.0066	0.2017
rs946824	1	243684019	C	0.859	-0.0206	0.0026	1.10E-15	0.8525	0.0104	0.0101	0.3058
rs947612	6	73738661	A	0.7516	-0.0116	0.0020	5.60E-09	0.7334	0.0020	0.0076	0.7924
rs9478671	6	155987825	G	0.2087	0.0120	0.0021	1.70E-08	0.2029	0.0088	0.0083	0.2869
rs9538162	13	59265043	C	0.4138	-0.0156	0.0018	4.80E-19	0.4355	-0.0037	0.0067	0.5850
rs9547153	13	85903717	G	0.3839	0.0098	0.0017	8.70E-09	0.3896	-0.0025	0.0068	0.7101
rs9688431	6	73922654	C	0.06034	-0.0231	0.0035	2.40E-11	0.0583	-0.0122	0.0140	0.3827
rs9816226	3	185834499	T	0.8199	0.0323	0.0021	1.60E-52	0.7939	-0.0018	0.0086	0.8373
rs9845966	3	13433158	G	0.5479	-0.0105	0.0017	2.50E-10	0.5448	0.0021	0.0066	0.7509
rs9951619	18	56882326	G	0.7643	0.0156	0.0020	1.40E-15	0.7451	0.0010	0.0078	0.8934
rs998732	19	19378671	G	0.1578	-0.0171	0.0022	2.00E-14	0.1579	0.0050	0.0092	0.5875
rs9989141	14	94006257	T	0.6387	0.0162	0.0017	3.60E-21	0.6326	0.0087	0.0069	0.2095

Abbreviations: SNP, single-nucleotide polymorphism; Chr, chromosome; Pos, position; EA, effect allele; EAF, effect allele frequency; SE, standard error; AF, atrial fibrillation.

Table S2. The combined effect estimators of the remaining 302 SNPs obtained by IVW after removing each SNP

SNP	OR	P	SNP	OR	P	SNP	OR	P
rs10009336	1.43	3.57E-34	rs17203016	1.42	1.54E-33	rs4818225	1.42	7.41E-34
rs1006896	1.43	1.25E-34	rs17238110	1.42	7.94E-34	rs4820408	1.42	5.80E-34
rs10132280	1.42	1.27E-33	rs17311369	1.43	4.05E-34	rs4858193	1.43	1.58E-34
rs10169594	1.42	5.86E-34	rs17399237	1.42	5.73E-34	rs4864201	1.42	1.22E-33
rs10197031	1.42	2.22E-33	rs17424296	1.42	8.20E-34	rs4880341	1.42	7.04E-34
rs10243319	1.43	2.79E-34	rs17425707	1.42	5.21E-34	rs4906908	1.42	9.42E-34
rs10247983	1.43	3.41E-34	rs17446257	1.43	3.11E-34	rs491711	1.42	6.97E-34
rs10248136	1.43	3.69E-34	rs17499593	1.42	5.55E-34	rs4929923	1.43	4.56E-34
rs10269783	1.43	2.19E-34	rs175165	1.42	1.03E-33	rs4936175	1.43	3.52E-34
rs1048932	1.43	4.60E-34	rs17535749	1.42	5.98E-34	rs4954638	1.43	4.32E-34
rs10742752	1.42	6.18E-34	rs17551974	1.42	1.24E-33	rs4986044	1.43	2.41E-34
rs10768994	1.43	2.65E-34	rs17636031	1.42	7.16E-34	rs538579	1.43	3.24E-34
rs10795422	1.43	1.49E-34	rs17663412	1.42	4.98E-34	rs543874	1.43	6.08E-34
rs10811871	1.42	5.06E-34	rs17710386	1.42	5.56E-34	rs559231	1.43	2.43E-34
rs10858334	1.43	4.07E-34	rs1830074	1.43	3.01E-34	rs577525	1.42	1.12E-33
rs10867256	1.42	4.75E-34	rs1836303	1.42	5.39E-34	rs592483	1.42	1.22E-33
rs10914462	1.42	4.95E-34	rs1843328	1.42	4.80E-34	rs6443750	1.43	3.29E-34
rs10920678	1.43	3.52E-34	rs1863652	1.42	4.93E-34	rs6448587	1.43	3.23E-34
rs10938397	1.42	9.34E-33	rs1884389	1.42	4.53E-34	rs6512302	1.42	6.74E-34
rs10942267	1.43	1.85E-34	rs1885728	1.42	4.86E-34	rs6545714	1.43	2.55E-34
rs10953740	1.42	2.43E-33	rs1891216	1.43	1.97E-34	rs6561943	1.42	5.74E-34
rs10968114	1.43	1.61E-34	rs1896767	1.42	7.54E-34	rs657452	1.43	1.50E-34
rs10984756	1.43	1.54E-34	rs1927790	1.43	1.42E-34	rs6587552	1.43	1.50E-34
rs11030618	1.42	5.35E-34	rs1937683	1.42	7.42E-34	rs6591407	1.42	5.60E-34
rs11105839	1.42	4.78E-34	rs1948080	1.42	4.95E-34	rs6593688	1.42	5.54E-34
rs11118308	1.43	1.82E-34	rs1982441	1.43	4.36E-34	rs6692586	1.43	1.67E-34
rs1112613	1.42	6.64E-34	rs1982725	1.43	3.83E-34	rs6764533	1.43	4.45E-34
rs11150911	1.42	8.54E-34	rs2007231	1.43	2.08E-34	rs6772756	1.42	8.25E-34
rs11165643	1.43	5.47E-34	rs200810	1.43	4.59E-34	rs6785245	1.43	1.53E-34
rs11170468	1.43	4.24E-34	rs2009416	1.43	3.91E-34	rs6804842	1.42	2.24E-33
rs11173522	1.42	5.66E-34	rs2051559	1.43	3.25E-34	rs6841761	1.42	1.08E-33
rs11185111	1.42	5.86E-34	rs208015	1.42	8.35E-34	rs685870	1.43	3.17E-34
rs11251352	1.43	2.72E-34	rs2124499	1.42	7.64E-34	rs7025938	1.42	2.02E-33
rs11505821	1.42	5.89E-34	rs2143253	1.43	3.22E-34	rs7037266	1.42	6.06E-34
rs11609659	1.42	6.38E-34	rs2162524	1.42	8.70E-34	rs705217	1.42	7.91E-34
rs11615578	1.43	2.58E-34	rs217671	1.42	5.69E-34	rs7138803	1.43	6.45E-34
rs11656076	1.42	9.06E-34	rs2228213	1.42	6.92E-34	rs7148846	1.43	2.12E-34
rs11672660	1.43	1.82E-34	rs2246012	1.42	8.06E-34	rs7172627	1.43	2.80E-34
rs11738695	1.42	1.10E-33	rs2283093	1.42	5.38E-34	rs7196720	1.43	2.74E-34
rs11739877	1.42	1.13E-33	rs2306537	1.42	1.30E-33	rs7334078	1.42	5.59E-34
rs11855853	1.43	3.63E-34	rs2317299	1.42	8.64E-34	rs7488867	1.43	2.88E-34
rs1187352	1.42	4.58E-34	rs2357760	1.42	1.90E-33	rs7519259	1.42	8.08E-34
rs11951673	1.43	4.55E-34	rs2361988	1.43	2.72E-34	rs7535528	1.43	3.64E-34
rs12033257	1.42	9.48E-34	rs2365389	1.43	5.73E-34	rs7550711	1.43	6.79E-34
rs12041258	1.43	2.01E-34	rs2367112	1.43	4.06E-34	rs7551507	1.43	3.33E-34
rs1218822	1.43	4.48E-34	rs2423668	1.43	2.01E-34	rs7599312	1.42	1.18E-33
rs12334877	1.43	4.18E-34	rs2425840	1.43	3.93E-34	rs7615297	1.43	4.16E-34

rs12364470	1.42	9.87E-34	rs2429150	1.43	4.32E-34	rs7637852	1.43	1.70E-34
rs12416812	1.43	2.55E-34	rs2479958	1.43	3.10E-34	rs765875	1.43	2.59E-34
rs1241986	1.42	5.85E-34	rs2481665	1.42	7.47E-34	rs7683836	1.42	6.87E-34
rs12422552	1.43	3.41E-34	rs2543132	1.43	2.26E-34	rs768840	1.43	4.19E-34
rs12546578	1.43	1.39E-34	rs2605603	1.42	9.97E-34	rs7694732	1.43	3.92E-34
rs12564992	1.43	4.09E-34	rs2608703	1.43	4.63E-34	rs7704281	1.42	1.10E-33
rs12629015	1.42	1.25E-33	rs262130	1.43	1.59E-34	rs7715256	1.42	1.05E-33
rs1266874	1.43	3.23E-34	rs2693826	1.42	8.39E-34	rs7730004	1.42	1.38E-33
rs12675063	1.43	3.87E-34	rs273504	1.42	6.94E-34	rs7730898	1.43	1.86E-34
rs1268065	1.42	1.17E-33	rs2791653	1.43	3.85E-34	rs774246	1.43	2.22E-34
rs12718572	1.42	1.07E-33	rs2832283	1.43	2.04E-34	rs7780752	1.42	5.06E-34
rs12762034	1.43	1.54E-34	rs2861683	1.42	2.11E-33	rs7788008	1.43	1.74E-34
rs1285997	1.42	9.50E-34	rs287104	1.42	5.48E-34	rs7811342	1.42	4.75E-34
rs12888545	1.43	2.35E-34	rs2875762	1.42	1.29E-33	rs7819514	1.42	9.01E-34
rs12905439	1.43	3.75E-34	rs2931434	1.43	3.55E-34	rs7826312	1.43	2.80E-34
rs12922346	1.42	5.71E-34	rs2943465	1.42	1.14E-33	rs7844647	1.43	3.88E-34
rs12939549	1.43	5.41E-34	rs294704	1.43	3.59E-34	rs7869771	1.43	2.39E-34
rs1296328	1.42	3.10E-33	rs3007105	1.42	9.51E-34	rs7871866	1.42	1.30E-33
rs13047416	1.43	3.00E-34	rs33500	1.42	1.31E-33	rs7899106	1.42	6.07E-34
rs13069244	1.42	5.65E-34	rs339991	1.42	1.00E-33	rs7925214	1.42	1.05E-33
rs13110266	1.42	1.12E-33	rs349088	1.43	2.90E-34	rs7998796	1.42	4.42E-34
rs13147390	1.43	3.01E-34	rs3731695	1.43	4.49E-34	rs8027205	1.42	5.01E-34
rs13174863	1.43	2.68E-34	rs3732084	1.42	6.11E-34	rs8036040	1.43	2.50E-34
rs13191362	1.42	1.03E-33	rs3736485	1.43	1.84E-34	rs806600	1.43	3.40E-34
rs1320903	1.43	2.30E-34	rs3749897	1.43	4.36E-34	rs8071182	1.43	2.74E-34
rs1321432	1.42	1.66E-33	rs3764835	1.43	3.26E-34	rs8090983	1.42	4.62E-34
rs13250058	1.43	2.04E-34	rs3772882	1.42	7.27E-34	rs8097783	1.42	1.83E-33
rs13263601	1.42	1.00E-33	rs3800229	1.43	1.09E-34	rs8123881	1.42	6.85E-34
rs1327259	1.42	5.76E-34	rs3800637	1.42	1.05E-33	rs8181823	1.43	1.86E-34
rs13287131	1.42	5.68E-34	rs3806114	1.43	2.48E-34	rs818524	1.43	3.75E-34
rs1330052	1.43	3.49E-34	rs3806572	1.43	2.02E-34	rs825688	1.43	4.17E-34
rs1365466	1.42	6.54E-34	rs3807645	1.42	5.56E-34	rs845084	1.42	5.71E-34
rs1371108	1.42	5.16E-34	rs380857	1.43	3.33E-34	rs852056	1.42	6.55E-34
rs1412235	1.42	9.93E-34	rs38314	1.42	5.38E-34	rs872281	1.42	6.82E-34
rs1430387	1.42	7.73E-34	rs3844598	1.43	1.89E-34	rs895330	1.43	5.16E-34
rs1431659	1.43	5.70E-34	rs3902951	1.42	5.58E-34	rs9294260	1.43	1.73E-34
rs1445652	1.42	9.60E-34	rs3904244	1.42	1.18E-33	rs930295	1.42	5.48E-34
rs1465900	1.42	9.24E-34	rs3935648	1.42	1.10E-33	rs9304665	1.43	9.20E-35
rs1476322	1.42	4.97E-34	rs3977755	1.43	3.45E-34	rs9362662	1.42	7.97E-34
rs1477199	1.42	1.75E-33	rs4072917	1.42	6.24E-34	rs9367368	1.43	3.93E-34
rs1492767	1.43	3.48E-34	rs4307239	1.43	1.43E-34	rs9408882	1.43	1.71E-34
rs1503526	1.43	4.58E-34	rs4310573	1.43	3.32E-34	rs946824	1.43	1.35E-34
rs1522569	1.43	1.69E-34	rs4358081	1.43	2.56E-34	rs947612	1.43	2.78E-34
rs1528435	1.42	1.27E-33	rs4430672	1.42	6.65E-34	rs9478671	1.42	5.63E-34
rs1535660	1.42	8.24E-34	rs4482463	1.42	6.42E-34	rs9538162	1.43	4.18E-34
rs1538247	1.43	3.00E-34	rs4518345	1.43	4.02E-34	rs9547153	1.43	2.70E-34
rs156201	1.43	2.11E-34	rs4556997	1.43	2.54E-34	rs9688431	1.42	5.23E-34
rs1624134	1.43	2.42E-34	rs4589691	1.43	3.59E-34	rs9816226	1.43	1.14E-34
rs1656377	1.42	8.65E-34	rs4639527	1.43	3.18E-34	rs9845966	1.43	2.65E-34

rs1681740	1.43	3.36E-34	rs4653017	1.42	4.76E-34	rs9951619	1.43	3.17E-34
rs16871902	1.42	5.58E-34	rs4671328	1.43	1.35E-34	rs998732	1.43	2.08E-34
rs17014375	1.42	8.46E-34	rs4722398	1.43	3.72E-34	rs9989141	1.42	7.55E-34
rs17033117	1.42	5.44E-34	rs4783830	1.42	9.59E-34	IVW	1.43	3.72E-34
rs17113297	1.42	1.25E-33	rs4786903	1.43	3.66E-34			
rs17119937	1.42	4.53E-34	rs4800191	1.43	1.95E-34			

Abbreviations: SNP, single-nucleotide polymorphism; OR, odds ratio; IVW, inverse variance weighted.
